# Visual interpretation, not SUV ratios, is the ideal method to interpret 18F-DOPA PET scans to aid in the cure of patients with focal congenital hyperinsulinism

**DOI:** 10.1371/journal.pone.0241243

**Published:** 2020-10-27

**Authors:** Pradeep K. Garg, Burton Putegnat, Lisa Truong, Courtney Reynolds, Irene Sanchez, Jonathan K. Nedrelow, John Uffman, Stephen J. Lokitz, Rachid Nazih, Sudha Garg, Paul S. Thornton

**Affiliations:** 1 Center for Molecular Imaging and Therapy, Biomedical Research Foundation, Shreveport, Louisiana, United States of America; 2 Cook Children’s Medical Center, Fort Worth, Texas, United States of America; Spedali Civili of Brescia, University of Brescia, ITALY

## Abstract

**Introduction:**

Congenital hyperinsulinism is characterized by abnormal regulation of insulin secretion from the pancreas causing profound hypoketotic hypoglycemia and is the leading cause of persistent hypoglycemia in infants and children. The main objective of this study is to highlight the different mechanisms to interpret the ^18^F-DOPA PET scans and how this can influence outcomes.

**Materials and methods:**

After ^18^F-Fluoro-L-DOPA was injected intravenously into 50 subjects’ arm at a dose of 2.96–5.92 MBq/kg, three to four single-bed position PET scans were acquired at 20, 30, 40 and 50-minute post injection. The radiologist interpreted the scans for focal and diffuse hyperinsulinism using a visual interpretation method, as well as determining the Standard Uptake Value ratios with varying cut-offs.

**Results:**

Visual interpretation had the combination of the best sensitivity and positive prediction values.

**Conclusions:**

In patients with focal disease, SUV ratios are not as accurate in identifying the focal lesion as visual inspection, and cases of focal disease may be missed by those relying on SUV ratios, thereby denying the patients a chance of cure. We recommend treating patients with diazoxide-resistant hyperinsulinism in centers with dedicated multidisciplinary team comprising of at least a pediatric endocrinologist with a special interest in hyperinsulinism, a radiologist experienced in interpretation of ^18^F-Fluoro-L-DOPA PET/CT scans, a histopathologist with experience in frozen section analysis of the pancreas and a pancreatic surgeon experienced in partial pancreatectomies in patients with hyperinsulinism.

## Introduction

Congenital hyperinsulinism (CHI) is characterized by abnormal regulation of insulin secretion from the pancreas causing profound hypoketotic hypoglycemia. It is the leading cause of persistent hypoglycemia in infants and children [1, 2]. The most common forms of CHI are caused by recessive mutations of either of the two genes encoding the β-cell ATP sensitive potassium channel (*ABCC8* and *KCNJ11*). Early detection and aggressive intervention is crucial to prevent long-term neurological complications [3]. Modulating the ATP-sensitive potassium channels of the beta cell through medical treatment with diazoxide in conjunction with glucagon, octreotide and/or nifedipine are some of the options to inhibit insulin secretion and in turn, control the hypoglycemia [4–9]. However, in a significant number of CHI patients, these treatments are not effective and sometimes associated with serious side-effects [3, 10–12]. In patients who have failed medical therapy and have two recessive mutations causing diffuse disease, surgical treatment with a 98% pancreatectomy is an alternate treatment option to modify the degree of hypoglycemia [13]. However, this approach often ends in either short-term (25%) or long-term diabetes (>95% by age 15) or pancreatic insufficiency [14, 15]. For those patients with a single paternally inherited mutation in *ABCC8* or *KCNJ11*, the surgical outcome depends on the ability to identify the precise location of the focal lesion within the pancreas. This enables the surgeon to remove the focal lesion with minimal resection of normal pancreatic tissue and minimize the long-term risk of diabetes and pancreatic insufficiency [16–19]. Therefore, it is imperative to identify patients with the focal versus diffuse forms of this disease for better treatment outcome. Conventional imaging using abdominal ultrasound, transesophageal ultrasound, computed tomography (CT) or magnetic resonance imaging (MRI) cannot localize focal lesions due to their inability to identify subtle morphological abnormalities that lack distortion of pancreatic structures, as these lesions are primarily functional lesions, although identifiable at a microscopic level by the pathologist [20]. Intraoperative ultrasound has been shown in some circumstances to identify the focal lesions when the probe is placed on the pancreas [21]. Dynamic imaging modalities, including pancreatic venous sampling or pancreatic arterial calcium stimulation methods, are invasive and technically challenging and not widely available [19, 22].

Recently, the non-invasive imaging of CHI-patients using positron emission tomography (PET) with ^18^F-Fluoro-L-DOPA has shown promising results [20, 23–25]. ^18^F-Fluoro-L-DOPA is a fluorinated analogue of L-dopamine, a crucial intermediate in the dopamine synthesis pathway and has been extensively used to image neuroendocrine tumors [26]. The ability of ^18^F-Fluoro-L-DOPA to image the pancreas resides in the neuroendocrine nature of pancreatic cells [1, 24, 27, 28]. The endocrine cells, located in the islet peripheries of pancreas, decarboxylate ^18^F-Fluoro-L-DOPA to dopamine through the action of aromatic amino acid decarboxylase enzyme (AADC) prior to its accumulation within the β-cells of the pancreas [29–31]. In recent years ^18^F-Fluoro-L-DOPA imaging has played an important role in the management of focal disease in patients with CHI [23, 24, 32–34].

Currently, there is ample evidence in the literature that the standard of care for patients with CHI who do not have genetic evidence of diffuse disease should include ^18^F-Fluoro-L-DOPA PET with CT or MRI imaging prior to surgery in those unresponsive to diazoxide [35]. In the centers using a multidisciplinary approach with ^18^F-Fluoro-L-DOPA PET/CT, cure rates in over 90% of focal patients can be expected. However, there is controversy about how to interpret the ^18^F-Fluoro-L-DOPA PET/CT scans, resulting in missed opportunities to cure focal disease because cases are overlooked depending on the criteria used to suspect focal disease. In addition, some centers use ^18^F-Fluoro-L-DOPA to determine whether patients have focal or diffuse disease and others use the scans to identify where focal lesions may be located. This subtle difference in approach has a significant bearing on outcomes. In this paper, we highlight the different mechanisms to interpret the ^18^F-Fluoro-L-DOPA PET scans and how this can influence outcomes.

## Methods

This study was approved by the Radiation Safety Committee and the Institutional Review Board of the Cook’s Children Health Care System, Fort Worth, Texas. Written informed consent was obtained from the legal authorized representative of each participating child prior to initiating the PET studies. Subjects were eligible for inclusion in this study if they had a diagnosis of CHI and genetic studies were either not done at the time of the scan or did not support a diagnosis of diffuse disease.

Nucleophilic fluorination method was used to obtain high specific activity ^18^F-Fluoro-L-DOPA for this study. The synthesis was performed using a previously described method with minor modification and by adapting the entire synthesis process to a GE FXn module (GE, Wisconsin, USA) [36, 37]. The ^18^F-Fluoro-L-DOPA was administered as part of an Investigational New Drug licensed study (IND # 119557). All patients are made NPO for the sedation and to allow us to control the glucose with a goal of getting the glucose level below the threshold for insulin secretion but above the risk of harm (60mg/dL to 75mg/dL) by adjusting the glucose infusion to minimize insulin secretion from the normal quiescent pancreas.

^18^F-Fluoro-L-DOPA was injected intravenously into the subject’s arm at a prescribed dose of 0.08–0.16 mCi (2.96–5.92 MBq/kg) with a mean injected dose of 1.35 ± 1.38 mCi (49.95 ± 51.06 MBq). The range of dose injected was 0.37–7.6 mCi (13.69–281.2 MBq). After acquiring a CT scan for attenuation correction purposes, three single-bed position PET scans (600 seconds each) centered on the abdomen were acquired at 20, 30, and 40 min in 3-D mode and in 19 patients an additional scan at 50 min post injection.

The PET/CT images were acquired on a Biograph-mCT PET/CT scanner (Siemens, Tennessee, USA). The imaging characteristics of this scanner have been described previously [38]. Iterative image reconstructions were performed to each PET image set using a fully 3-D Ordinary Poisson Ordered Subset Expectation Maximization (3D OP OSEM) algorithm with measured attenuation correction and a modeled scatter correction in addition to random correction using the delayed correction method [39, 40].

The assembled PET images were co-registered with the CT images to further aid in localizing the pancreas and its vascular supply. Overall radioactivity accumulation and the uptake pattern was noted on the radiologist report. Visual and quantitative analysis of PET images were performed looking for two distinct ^18^F-Fluoro-L-DOPA accumulation patterns in the pancreas. A distinctly visible hot-spot that was classified as focal disease and a relatively uniform or somewhat patchy uptake pattern throughout the pancreas was classified as diffuse.

The image set for each patient was analyzed in several different ways after the completion of the PET scan by a radiologist. First, visual analysis of the PET scans was performed and each scan was reviewed in all three planes and in maximum intensity projection 3D (MIP) views. The PET scans were further subjected to quantitative analysis method using PMOD (PMOD Technologies, Zurich, Switzerland). The averaged standardized uptake value (SUV a) was calculated by using the mean radioactivity concentration values normalized to body weight and injected dose amount, using the formula SUV a = A/(IA X BWt), where A is the activity concentration within the volume of interest (Bq/mL), BWt is the body weight of patient and IA is the injected activity in Bq. All SUVs were decay corrected for F-18 radionuclide half-life. In addition, maximum standardized uptake value (SUV M) and the second highest standardized uptake value (SUV 2^nd^M) for the pancreas in each PET scan was also obtained. SUV R is the ratio of SUV M /SUV 2^nd^M.

Due to the variability in the literature that currently exists regarding the optimal method for interpreting the PET/CT scans, four methods were examined and compared for accuracy: 1.) the visual inspection method 2.) SUV A ≥2 using SUV M/SUV a where ≥2 was classified as the cut-off point for focal disease 3.) SUV R≥1.3 using SUV M/2^nd^M with a ratio cut-off of ≥ 1.3, and 4.) SUV R ≥1.5 using SUV M/2^nd^M with a ratio cut-off of ≥ 1.5.

At surgery, three biopsies, one each from the head, body and tail of the pancreas, were sent for frozen section analysis. Frozen sections were stained with H&E and examined under the microscope. Focal disease was defined as three normal random biopsies of the pancreas showing suppressed insulin secreting cells, whereas diffuse as three abnormal biopsies containing islets with nucleomegaly. Upon confirming focal disease, the surgeon searched the area defined by the PET scan with the increased uptake and resected suspicious tissue. Histologically the focal lesions showed a localized proliferation of endocrine tissue. The focal lesions of CHI are typically admixed with variable amounts of native ductal structures and acinar tissue. Nucleomegaly, while present in some lesions, is not as reliably present as it is in diffuse disease of CHI. Nucleomegaly is defined as the identification of an increased number of islet cell nuclei which are at least three times the size of adjacent islet cells nuclei [41] and/or four times the size of adjacent acinar cell nuclei [42]. For biopsies defined as diffuse, the surgeon performed a 98% pancreatectomy. Focal lesions also demonstrate loss of nuclear reactivity to p57^kip2^ immuno-histochemistry as a result of loss of heterozygosity of 11p15, which includes the maternally expressed growth suppressor *CDKN1C* that encodes p57^kip2^, whereas nuclei of diffuse disease show retained nuclear immunoreactivity for p57^kip2^ immunohistochemistry. These studies for immunoreactivity can only be performed on paraffin embedded tissue and are not available at the time of frozen section.

## Results

**Fig 1:**
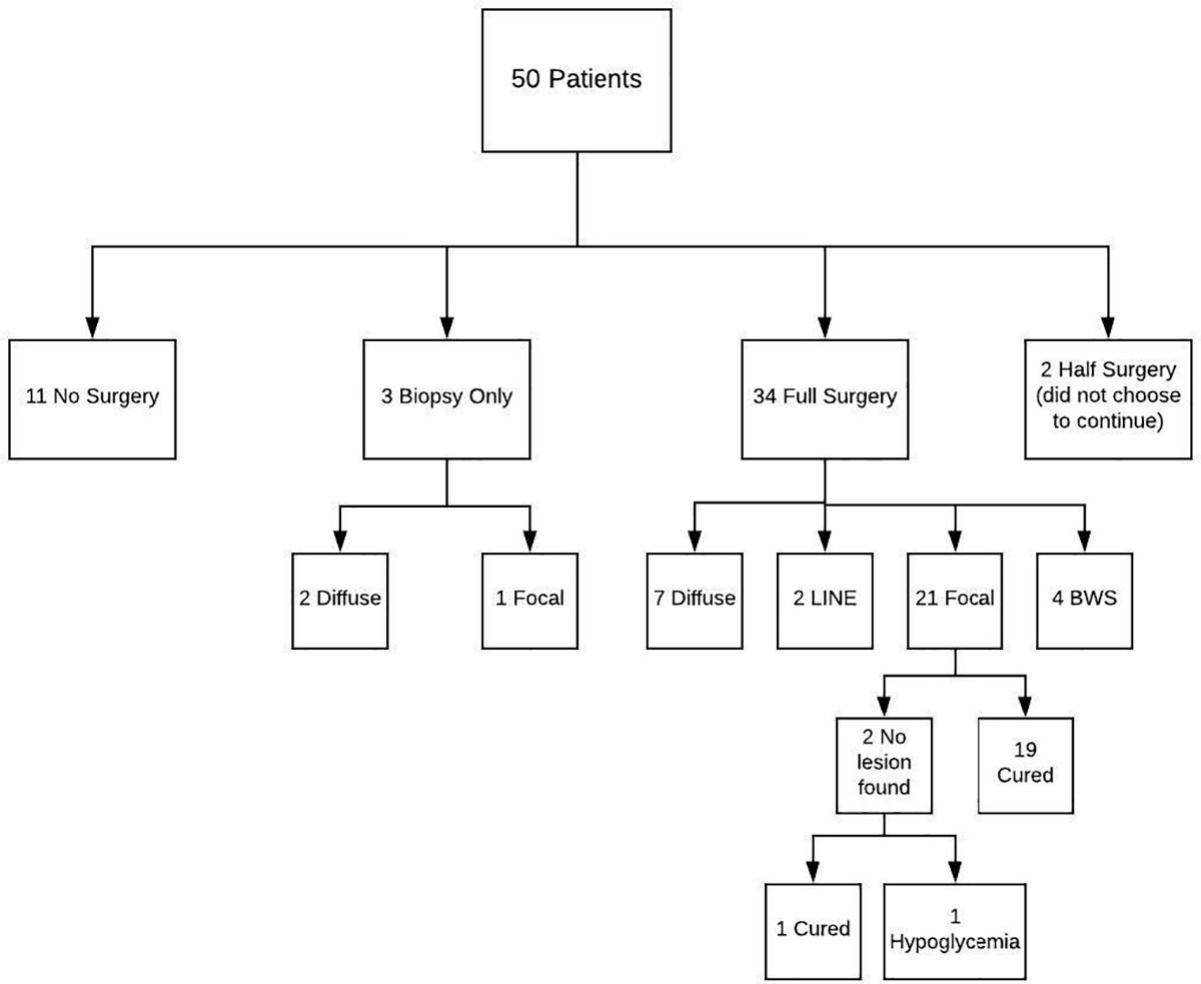
Flowchart of 50 subjects.

A total of 50 patients with CHI were studied using ^18^F-Fluoro-L-DOPA positron emission tomography (PET) (Fig 1). The demographics are shown in Table 1. There were six patients whose first episode of hypoglycemia and hyperinsulinism was first identified after one year of age (Patient numbers 5, 7,11, 31, 45, 46 in Table 2). Patients 5, 7 and 11 had negative genetic testing and did not undergo surgery as the PET scans did not have evidence of focal disease and the patients were adequately controlled on medication. Patient 31 has a Paternal *ABCC8* mutation and had focal disease and was cured with local resection of the lesion. Patients 45 and 46 had negative mutations and had LINE pathology and were cured after partial pancreatectomy. In addition, there were two patients who had hypoglycemia diagnosed after one year of age, but a retrospective chart review showed that they had hypoglycemia at birth which was not considered pathological at the time (patients 12 and 24 in Table 2). Patient 12 had mosaic expression of GK and only had biopsies of pancreas (done while inserting G Button and before results of genetic tests were completed) showing diffuse disease and has persistent hypoglycemia moderately controlled by diazoxide. Patient 24 also had a Paternal ABCC8 mutation, focal disease on histology and was cured with focal resection of the lesion.

**Table 1:** Demographics of 50 subjects.

Female (%)	19 (38)
Race (%)	
White	40 (80)
Black	5 (10)
Asian	2 (4)
Other	3 (6)
Hispanic (%)	17 (34)
Birth Weight (grams)	
n	48
Median	3,512.9
Range	1,925.0–5,070.0
Age at Hypoglycemia Onset (days)	
n	50
Median	0.0
Range	0–1,461
Age at HI DX (days)	
n	49
Median	22
Range	0–3,478
Age at Imaging (days)	
n	50
Median	125.5
Range	14–4,114

**Table 2:** Individual subject data.

	SUV A ≥2	SUV M/2^nd^M	Visual	SUV A ≥2	SUV R ≥1.3	SUV R ≥1.5	Pathology	Mutations	Glycemic Outcome
1	1.90	1.08	D	D	D	D		**ABCC8**: c.2506C>T, p.(Arg836*); c.1462A>C, p.(Thr488Pro)	hypoglycemia
2	1.31	1.15	D	D	D	D		**ABCC8: c.892 C>T, p.(Arg298Cys)**	hypoglycemia
3	1.89	1.06	D	D	D	D		Negative	hypoglycemia
4	2.25	1.26	D	F	D	D		KCNJ11: c.179T>C, p.(Phe60Ser); c.973C>A, p.(Arg325Ser)	hypoglycemia
5	1.98	1.1	D	D	D	D		Negative	hypoglycemia
6	3.62	1.29	D	F	D	D		Negative	hypoglycemia
7	2.18	1.15	D	F	D	D		Negative	hypoglycemia
8	1.97	1.07	D	D	D	D		Negative	hypoglycemia
9	2.62	1.14	D	F	D	D		ABCC8: c.4460A>G, p.(Gln1487Arg)	hypoglycemia
10	2.13	1.23	D	F	D	D		Negative	hypoglycemia
11	2.26	1.08	D	F	D	D		Negative	hypoglycemia
12	2.18	1.06	D	F	D	D	D	*GCK*: *c*.*1364_1366dup*, *p*.*Ala455dup*	hypoglycemia
13	2.44	1.13	D	F	D	D	D	*ABCC8*:*c*.*3455C>A*, *p*.*(Ale1152Asp);* KCNJ11 c.174C>A, p.(Asp58Glu)	hypoglycemia
14	1.44	1.27	F	D	D	D	F^^^	ABCC8:c.1792C>T, p.(Arg598*)	hypoglycemia
15	3.25	2.01	F	F	F	F	F^^^	**ABCC8:c.4369G>A, p.(Ala1457Thr)**	hypoglycemia
16	2.67	1.35	F	F	F	D	F^^^	*ABCC8*:*c*.*3509del*, *p*.(Leu1170Argfs*38)	hypoglycemia
17	1.78	1.08	D	D	D	D	D	Negative	hypoglycemia
18	1.79	1.27	D	D	D	D	D	ABCC8:c.1252T>C, p.(Cys418Arg)	hypoglycemia
19	2.73	1.61	F	F	F	F	D	**ABCC8:c.1176+2T>C, p.?**	hypoglycemia
20	2.55	1.05	F	F	D	D	D	ABCC8: c.928G>A, p.(Asp310Asn), c.4178G>A, p.(Arg1393His)	hypoglycemia
21	1.96	1.16	F	D	D	D	D	ABCC8: c.2506C>T, p.(Arg836*), c.2989dup, p.(Trp997Leufs*117)	hypoglycemia
22	2.11	1.31	D	F	F	D	D	*ABCC8*:*c*.*4460A>G*, *p*.*(Gln1487Arg)*	hypoglycemia
23	2.19	1.49	F	F	F	D	D	ABCC8:c.2797C>G, p. (Arg933Gly)	hypoglycemia
24	3.21	1.58	F	F	F	F	F	**ABCC8:c.3989-9G>A, p.?**	cured
25	2.29	1.70	F	F	F	F	F	ABCC8:c.2506C>T, p.(Arg836*)	cured
26	2.11	1.16	F	F	D	D	F	Negative	cured
27	2.39	1.35	F	F	F	D	F	*ABCC8*:*c*.*2921-3C>G*, *p*.?*; c*.*3018C>G*, *p*.*(Ser1006 =)*	cured
28	3.41	3.09	F	F	F	F	F	*ABCC8*:*c*.*2414G>A*, *p*.*(Cys805Tyr)*	cured
29	2.11	1.11	D	F	D	D	F	ABCC8:c.2992C>T, p.(Arg998*)	cured
30	2.22	1.27	F	F	D	D	F	**ABCC8:c.2506C>T**, p.(Arg836*)	cured
31	2.95	1.07	F	F	D	D	F	**ABCC8:c.4119+1G>C, p.?**	cured
32	2.24	1.76	F	F	F	F	F	*ABCC8*:*c*.*3574del*, *p*.(Asp1192Metfs*16)	cured
33	2.98	1.21	F	F	D	D	F	ABCC8:c.2295_2307delinsAA, p.(Arg766Serfs*21)	cured
34	3.29	2.37	F	F	F	F	F	ABCC8:c.1254_1284dup, p.(Met429*)	cured
35	2.56	1.15	F	F	D	D	F	**ABCC8:c.4376T>G, p.(Leu1459Arg)**	cured
36	2.95	2.55	F	F	F	F	F	**ABCC8:c.563A>G, p.(Asn188Ser)**	cured
37	2.80	1.42	F	F	F	D	F	**KCNJ11:c.944T>C, p.(Phe315Ser)**	cured
38	2.83	1.24	F	F	D	D	F	**KCNJ11:c.776A>G, p.(His259Arg)**	cured
39	3.05	1.91	F	F	F	F	F	ABCC8:c.1634del, p.(Phe545Serfs*2)	cured
40	2.29	1.71	F	F	F	F	F	*ABCC8*: *c*.*221G>A*, *p*.*(Arg74Gln)*	cured
41	2.84	1.29	F	F	D	D	F	ABCC8: c.4306C>T, p.(Arg1436*)	cured
42	2.23	1.11	F	F	D	D	F	*KCNJ11*: *c*.*776A>G*, *p*.*(His259Arg)*	cured
43	1.54	1.36	F	D	F	D	F^^^	**ABCC8: c.4376T>G, p.(Leu1459Arg)**	hypoglycemia
44	2.14	1.44	F	F	F	D	F^^^	Negative	cured
45	1.62	1.09	F	D	D	D	LINE	Negative	cured
46	2.52	1.14	D	F	D	D	LINE	Negative	cured
47	2.27	1.11	D	F	D	D	BWS	**ABCC8:c.2390G>A, p.(Arg797Gln)**	hypoglycemia
48	2.73	1.53	F	F	F	F	BWS	*ABCC8*:*c*.*2857C>T*, *p*.(Gln953*)	hypoglycemia
49	2.98	1.22	F	F	D	D	BWS	Negative	hypoglycemia
50	2.19	1.4	F	F	F	D	BWS	ABCC8:c.1879del, p.(His627Metfs*20)	hypoglycemia

^^^No focal lesion found.

F = focal.

D = diffuse.

Mutation in bold = Paternally inherited.

Mutation italicized = Non-maternally inherited, father not tested.

The individual data regarding the ^18^F-Fluoro-L-DOPA PET/CT scans, SUV ratios, pathology results and genetics are shown in Table 2. Eleven patients (Table 2, patients 1–11) declined surgery and chose medical management. Typically, this was because genetics revealed diffuse disease after the PET scan was performed or because they were well enough medically controlled, and the PET scan did not suggest focal disease. Three patients had biopsies only (patients 12–14). PET scans for two of those patients suggested diffuse and they wished to confirm while having gastric button placed. The third patient had a focal lesion on the PET scan and biopsies were normal, but the patient developed severe portal hypertension secondary to umbilical catheter placement in the newborn period, making surgery to remove the lesion unsafe due to extensive venous engorgement of the pancreas. Two patients (patients 15 &16) had lesions in the head of the pancreas based on the PET/CT scans but declined consent to remove the head of the pancreas and perform a Roux-en-Y pancreatico-jejunostomy. All 16 patients were discharged with hypoglycemia managed medically. Thirty-four patients had full surgery (patients 17–50). Of these, 7 cases demonstrated histology consistent with the diffuse form of CHI (patients 17–23). None of these patients went home on Insulin, one on no medication, and six required medical treatment for ongoing hypoglycemia. Twenty-one of the patients had classical focal disease (patients 24–44), 2 patients had localized Islet cell nuclear hypertrophy (LINE) (patients 45–46) and 4 had extensive focal lesions with islet hypertrophy characteristic of Beckwith-Wiedemann Spectrum (BWSp) (patients 47–50). None of these four patients had outward clinical findings suggestive of BWSp, and all had negative genetic testing for BWSp in blood but two had positive tests in frozen pancreatic tissue from the lesion. The diagnosis was made based on the histological findings and confirmed in two with tissue genetics. The remaining two did not have tissue genetics performed but had independent pathological confirmation of our tissue findings (pathology lab at Children’s Hospital of Philadelphia). Out of the 21 subjects with focal CHI who had complete surgery, two subjects showed no focal lesion during surgery, one was cured (Table 2 patient 44) and the other remained hypoglycemic (Table 2 patient 43). Both had PET scans suggestive of head lesions and both had complete head resections with Roux-en-Y pancreatico-jejunostomy. The remaining 19 of the focal CHI subjects were cured with surgery in addition to the 2 patients with localized islet nuclear enlargement (LINE) pathology resulting in an overall cure rate of 95.6% in those patients with potential for cure.

For analysis of the accuracy of the different interpretation techniques, only the subset of patients who had both histology and PET/CT scans are included. Because four of the patients had BWSp and their histology matches neither the focal nor diffuse pattern, for the purpose of this paper they were not included. However, in routine clinical practice, the diagnosis of BWSp is often made based on the histological findings (extensive overgrowth of the pancreas with larger irregular shaped islets) and there may be subtle clues on the PET scan. Thus 33 patients were included in the analysis that follows.

The results of each method reported as sensitivity, specificity, positive predictive value (PPV) and negative predictive value (NPV) are shown in Table 3. The sensitivity of the method is the likelihood that all who had focal disease were correctly identified as focal by the PET scan. The specificity shows that of those with diffuse disease the percentage predicted by the PET scan as diffuse. The positive predictive value is the number of patients predicted to be focal by the PET who were actually focal, and the negative predictive value was the number of those predicted to be diffuse that actually were diffuse.

**Table 3:** Value of ^18^F-Fluoro-L-DOPA PET scan in identifying focal.

Method				
	Sensitivity	Specificity	PPV	NPV
Visual Interpretation	95.8	55.6	85.2	83.3
SUV A ≥ 2	91.7	33.3	78.6	60
SUV R ≥ 1.3	58.3	66.7	82.3	37.5
SUV R ≥ 1.5	37.5	88.9	90	34.8

In five patients (patients 26, 45, 29, 30 and 38), we found that when using the visual method, we identified areas of increased uptake that were the focal lesion but were not the highest SUV in the pancreas. Their data are shown individually in Table 4 and the SUV of the area where the focal lesion was found in shown in bold. In these cases, the SUV ratio is reported as SUV M/2^nd^M but in 4 of the 5 cases the lesion was the second highest SUV, and in one (patient 29) was the third highest. In all five, focal lesions were found by the surgeon looking at the area of increased uptake and identifying the tissue, visually and by manual palpation that suggested a focal lesion. In each case, the tissue was resected with minimal loss of normal tissue and all five patients were cured. We did not attempt to remove the area with the highest SUV in these five cases. In all cases, the head of the pancreas had the highest uptake.

**Table 4:** Individual SUV values in patients with mismatch between visual findings and peak SUV and location of lesion.

SUV M/2^nd^M																
ID		20 min				30 min				40 min			Visual Interpretation	Focal Lesion Location	Pathology	Outcome
	Head	Body	Tail	Ratio	Head	Body	Tail	Ratio	Head	Body	Tail	Ratio				
26	4.74	2.87	**4.39**	1.08	4.6	2.37	**3.98**	1.16	3.52	2.09	**3.65**	1.12	focal	tail	focal	cured
45	3.27	2.24	**3.12**	1.05	3.07	1.83	**2.93**	1.05	2.79	1.96	**2.55**	1.09	focal	tail	line	cured
29	5.1	**4.64**	4.86	1.05	4.99	**4.62**	4.62	1.08	4.81	**4.32**	4.3	1.11	diffuse	body	focal	cured
30	5.41	**4.25**	3.27	1.27	5.24	**4.14**	3.23	1.27	3.76	**3.64**	1.98	1.03	focal	body	focal	cured
38	7.51	4.45	**6.63**	1.13	7.42	3.99	**5.97**	1.24	6.35	3.43	**5.15**	1.23	focal	tail	focal	cured
19	6.72	**4.64**	3.16	1.45	6.08	**3.77**	2.84	1.61	4.77	**3.35**	2.4	1.42	focal body		diffuse	hypoglycemia
20	7.02	**7.35**	5.9	1.05	6.58	**6.87**	5.5	1.04	6.37	**6.15**	4.75	1.04	focal body		diffuse	hypoglycemia
21	3.71	**3.8**	2.44	1.02	3.69	**3.17**	2.44	1.16	2.78	**3.13**	2.18	1.13	focal body/tail		diffuse	hypoglycemia
23	**5.32**	3.57	2.73	1.49	**4.82**	3.34	2.26	1.44	**4.79**	3.33	2.34	1.44	focal head		diffuse	hypoglycemia

Bolded SUVs represent the location of focal lesion in patients 26,45,29,30,38 or in patients 19, 20, 21, 23 bolded SUV represents the area we thought was focal but pathology was diffuse.

**Fig 2:**
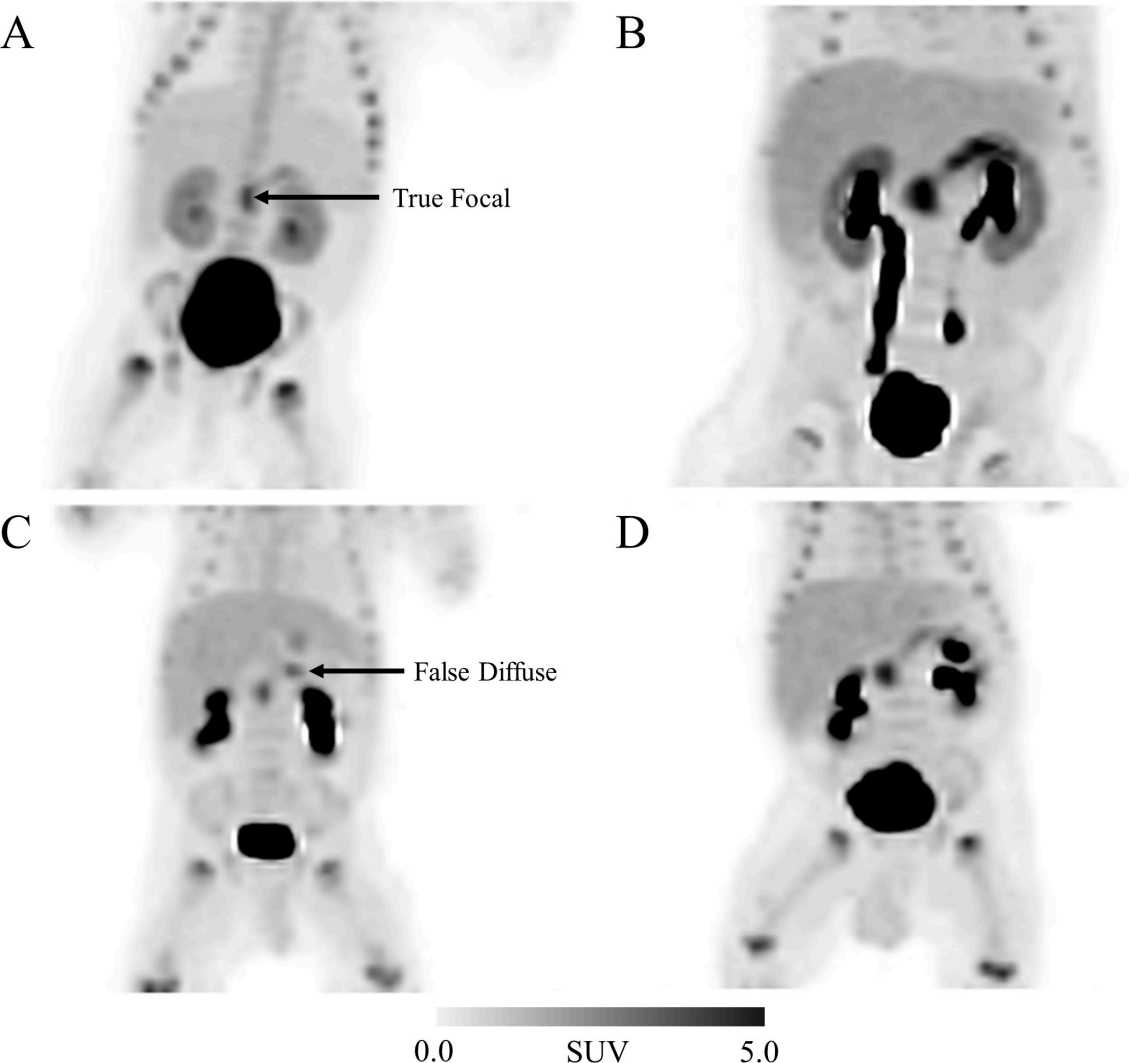
Maximum Intensity Projections (MIP) images of four subjects prepared from the image set acquired 30 minutes post [^18^F]Fluoro-L-DOPA administration. The four panels represent the following scenarios relating to the imaging analysis presented here and pathological results: A) focal disease confirmed pathologically, ID # 25, B) diffuse disease confirmed pathologically, ID #13, C) disagreement between imaging (diffuse) and pathology (focal), ID # 26, and D) disagreement between imaging (focal) and pathology (diffuse), ID #19.

In four cases with proven diffuse disease (patients 19, 20, 21, 23), we interpreted the visual reading to be focal based increased uptake in the head, body, body and head respectively (SUV shown in bold in Table 4). The two patients that we called as having head lesions had SUV M/2^nd^M values of 1.61 and 1.49 (highest SUV in the head) and the two we interpreted as body focal lesions had highest SUV measurements in the body but had SUV M/2^nd^M of 1.05 and 1.16. In these two cases, we interpreted focal because we expect the head to be the highest uptake as it often is even in diffuse case. Biopsies however indicated diffuse in all four, and 98% pancreatectomies were performed with outcome being persistent hypoglycemia requiring medical treatment. Maximum Intensity Projection (MIP) images generated from PET/CT scans from select representative four patients are presented in Fig 2.

We also analyzed the sensitivity and specificity of a single paternal mutation or a single nonmaternal mutation in *ABCC8/KCNJ11* to predict if a subject had focal disease. Of the 39 patients in whom we had a combination of histology and genetics one had GK HI, 2 had LINE pathology and 5 had Beckwith Wiedemann Spectrum and were excluded from the genetic sensitivity and specificity of the determination of focal or diffuse. This left 31 patients of whom 24 had focal and 7 had diffuse. The sensitivity of a single paternal mutation or a single nonmaternal mutation in *ABCC8/KCNJ11* to identify focal was 92%, the specificity was 71% and the positive predictive value was 92% and the negative predictive value was 71%.

## Discussion

CHI is classified either as transient or persistent, and in those with persistent disease, sub-classified as diazoxide sensitive or resistant. The majority of diazoxide resistant patients have mutations in the K_ATP_ channel encoded by the *ABCC8* and *KCNJ11* genes. Current diagnostic algorithms suggest that one further investigates diazoxide resistant patients with genetic testing and ^18^F-Fluoro-L-DOPA PET/CT or MRI because both tests can give an indication of the likelihood of having either focal or diffuse disease. However, only the ^18^F-Fluoro-L-DOPA PET/CT scan could localize the focal lesion, and thus allow the surgeon to re-sect the lesion and cure the patient. Interpretation of the ^18^F-Fluoro-L-DOPA PET/CT may be difficult and several methods have been proposed. One method is visual inspection in 3D mode of the maximum intensity projection (MIP) image (Fig 2). Laje et al describe the focal lesion as “bright signal over a darker background” [43]. Hardy et al.’s description of focal disease is as follows: “the image was considered positive for focal adenomatosis when the uptake of the radiotracer in a part of the pancreas was visually higher than the uptake in the remaining pancreatic tissue” [34]. Others have used quantitative analysis looking the ratio of activity in the lesion to the background activity. Thus, one can look at the ratio of SUV M to background or to average or to SUV 2^nd^M. Ratios of ≥1.5 or ≥1.3 have been used [33, 44]. Most metanalysis of accuracy of ^18^F-Fluoro-L-DOPA to diagnose and localize do not give final recommendations on the best way to interpret the scans. With the recent trend of not operating on diffuse patients gaining more traction due to the high likelihood of diabetes following 98% pancreatectomy (42% by age 8 and 91% by age 14), it is very important to determine who has focal and who has diffuse disease [45]. Decisions may be made not to operate on patients because the PET scan looks diffuse and this could result in missing the opportunity to cure patients with focal disease.

Therefore because of a lack of guidance in the literature as to the optimal method to interpret the ^18^F-Fluoro-L-DOPA PET/CT scan we undertook this study to compare the sensitivity, specificity, PPV and NPV of 4 different techniques. We found that no one technique had the best score in each outcome. We found that visual interpretation provided the best sensitivity but the ratio of the SUV M to the SUV 2^nd^M of greater than 1.5 (SUV R ≥1.5) provided the best PPV. This also provided the best specificity. However as seen in Table 4 the focal lesion is not always the area with the highest uptake value in the pancreas. In our experience in 23 patients with either classical focal adenomatosis or focal LINE pathology, the focal lesion was not in the area of the highest uptake values in 5 patients. Thus, in retrospective analysis of the PET scan, the ratio of the SUV of what turned out to be focal lesion to the highest area of uptake was less than 1. In prospective analysis, the ratio of the SUV M to SUV 2^nd^M would in all cases of indicated diffuse disease being less than 1.3 and thus might have led a multidisciplinary team to determine that this patient should not have surgery. In this case, had we deferred surgery because of a concern that this was diffuse disease we would have missed the opportunity to cure 22% of focal patients.

Our recommendation for interpreting ^18^F-Fluoro-L-DOPA PET/CT scans is as follows: first the image should be visualized using the MIPS image in three-dimensional views (Fig 2). Then specific uptake values of the head, body, and tail should be determined. Areas of increased uptake should be highlighted, and their anatomical position confirmed with the fused CT or MRI image. If the radiologist believes the patient has diffuse disease based on the SUV ratios, they will report that overall they believe the image shows SUV M: SUV 2^nd^M uptake of less than 1.3 (SUV R ≤ 1.3) suggesting diffuse disease, but that areas of increased uptake are located in either the head, body, or tail and that if at biopsy focal disease is found a certain area should be investigated first. In our experience, the results of the PET scan should not be used to determine whether surgery is performed or not, but rather, patients who need to go to surgery should have PET scan performed or those who have genetic evidence to suggest that focal disease is more likely should also have the PET scan performed. If focal disease is detected by biopsy subjected to frozen section histology, then the surgeon has a GPS roadmap through PET/CT scan to guide him to the lesion location. In addition, it is critical to have a highly experienced pancreatic surgeon who will look at all the areas of interest on the PET scan prior to removing the area of the highest uptake with careful visual inspection and manual palpation. In most cases they will be able to get clues as to the location of the lesion using these techniques when they are guided into the general area by the PET scan. It is for these reasons we recommend that patients who have diazoxide resistant CHI are treated in centers with dedicated multidisciplinary team comprising of at least a pediatric endocrinologist with a special interest in CHI, a radiologist experienced in interpretation of ^18^F-Fluoro-L-DOPA PET/CT scans, a histopathologist with experience in frozen section analysis of the pancreas and a pancreatic surgeon experienced in partial pancreatectomies in patients with CHI.
